# Thinking Beyond the Device: An Overview of Human- and Equity-Centered Approaches for Health Technology Design

**DOI:** 10.1146/annurev-bioeng-081922-024834

**Published:** 2023-04-17

**Authors:** Natalia M. Rodriguez, Grace Burleson, Jacqueline C. Linnes, Kathleen H. Sienko

**Affiliations:** 1 Weldon School of Biomedical Engineering, College of Engineering, Purdue University, West Lafayette, Indiana, USA; 2 Department of Public Health, College of Health and Human Sciences, Purdue University, West Lafayette, Indiana, USA; 3 Regenstrief Center for Healthcare Engineering, Purdue University, West Lafayette, Indiana, USA; 4 Design Science, College of Engineering, University of Michigan, Ann Arbor, Michigan, USA; 5 Department of Mechanical Engineering, College of Engineering, University of Michigan, Ann Arbor, Michigan, USA

**Keywords:** health technologies, human-centered design, equity-centered engineering, contextual investigation, community engagement, stakeholder engagement

## Abstract

A shift in the traditional technocentric view of medical device design to a human-centered one is needed to bridge existing translational gaps and improve health equity. To ensure the successful and equitable adoption of health technology innovations, engineers must think beyond the device and the direct end user and must seek a more holistic understanding of broader stakeholder needs and the intended context of use early in a design process. The objectives of this review article are (*a*) to provide rationale for the need to incorporate meaningful stakeholder analysis and contextual investigation in health technology development and biomedical engineering pedagogy, (*b*) to review existing frameworks and human- and equity-centered approaches to stakeholder engagement and contextual investigation for improved adoption of innovative technologies, and (*c*) to present case study examples of medical device design that apply these approaches to bridge the gaps between biomedical engineers and the contexts for which they are designing.

## 1. INTRODUCTION

Health technology innovation is growing at an unprecedented pace, yet the processes for successful implementation, adoption, and diffusion of new medical devices remain poorly understood and often exclude meaningful consideration of intended end users, stakeholders, and the broader communities and contexts in which they are situated. Often referred to as the translation gap (1) or implementation gap (2), this chasm between biomedical innovators and the people who need their innovations has created myriad barriers in translating research from the bench to clinical implementation at the bedside to delivery to communities most in need.

Merely meeting technical specifications is insufficient to ensure the success of a new technology (3). Successful medical device innovation requires investigation of end-user and broader stakeholder contexts and incorporation of those context-specific needs into design processes. Failure to incorporate relevant contextual information has been shown to lead to multiple medical device failures (4, 5). Garvin’s eight basic dimensions for a manufactured product (i.e., performance, features, reliability, conformance, durability, serviceability, aesthetics, and perceived quality) (6) have been cited in some engineering design texts (e.g., 7) and have served as a guide for designers when developing requirements. Accessibility, availability, accommodation, affordability, and acceptability have been considered in health policy, health services research, and health technologies, including medical devices (8, 9). Additionally, requirements related to usability [e.g., ergonomic attributes, required mental effort from users, and characteristics of user interaction (10)] and feasibility have been emphasized during design processes to date. However, comprehensive evaluations of human-centered needs, community priorities, and broader contextual constraints that also inform critical device requirements have been inconsistently considered, and, in some cases, not considered at all (11).

Effective contextual investigation requires engagement of the various individuals, organizations, and entities that either affect or are affected by the design problem at hand. Different stakeholders hold different forms of knowledge and perspectives that, when taken together, culminate in a more holistic understanding of a device’s development and use case. In addition to considering intended end users, literature recommends the incorporation of local and national governmental stakeholders, locally trusted nongovernmental organizations, and applicable implementation and design expertise (12,13). Engaging a broad set of stakeholders means that designers must use an array of user engagement techniques that are context appropriate by considering power dynamics, cultural norms, expectations and priorities, and general access to these groups or individuals. What questions designers ask and how they present these questions to stakeholders matter on the basis of who is being engaged and in what setting (14, 15).

In biomedical engineering (BME) training and practice, technical and scientific knowledge has been historically prioritized over other community-focused and interdisciplinary knowledge required for health impact (16). However, in the United States, the Patient Protection and Affordable Care Act of 2010 promoted advancement in patient care and equitable, quality solutions tailored to unique clinical situations and patient priorities (17). Recently, the coronavirus disease 2019 (COVID-19) pandemic has also brought to light needs for equitable technology development and distribution and has created new opportunities for patient-centered biomedical innovation. From accelerating the development and evaluation of vaccines and rapid diagnostic tests to catalyzing a cultural shift in healthcare delivery with broadened access through self-testing and telehealth, COVID-era innovation is relevant and needed, and it can be leveraged for other health conditions. Unfortunately, COVID has also magnified the persistent social and contextual inequities that lead to barriers to access and use of health innovations (18) and has further emphasized that in health and medicine, technological innovation alone is inadequate without incorporation of social, political, and cultural contextual considerations (19–21). In response, there has been increased emphasis on the engagement of communities and diverse stakeholders to understand and address the multilevel challenges to the design, implementation, adoption, and diffusion of health technologies (22).

A growing commitment to health equity and community engagement is now reflected in many major federal and global initiatives, including clinical and translational science programs, minority health and disparities research funding, regulatory approval requirements, and targeted programs such as the National Institutes of Health’s Rapid Acceleration of Diagnostics for Underserved Populations program. The World Health Organization (WHO) has also recently launched the WHO Health Innovation for Impact program, acknowledging that scaling up emerging technologies and health innovations requires a better understanding of barriers to implementation and uptake to reach the most vulnerable populations. Recent literature advocates that technology designers possess key skills, such as contextual comprehension and analysis, cross-cultural humility, and stakeholder analysis and engagement, particularly when developing solutions in global health contexts to address both the technological and societal aspects of creating innovative tools to address health disparities (23, 24).

A clear understanding of end users, communities, and broader stakeholder needs within their sociocultural, political, economic, and environmental contexts is critical to the successful and equitable uptake, adoption, and diffusion of health technology innovations. Numerous models and methods across engineering design literature exist to help designers incorporate this context-specific information into design processes. Biomedical engineers are poised to help bridge the gap in health technology implementation by encompassing more holistic approaches (e.g., human-centered, participatory, contextual, and equity-centered) in the design of real-world solutions that people will actually be willing and able to use. This review presents diverse strategies and examples in medical device innovation to help guide engineers in investigating contextual factors and engaging broad groups of stakeholders that inform appropriate technology design. The objectives of this article are: (*a*) to provide rationale for the need to incorporate meaningful stakeholder engagement and contextual investigation in health technology development and BME pedagogy, (*b*) to review existing frameworks and human- and equity-centered approaches to contextual investigation for improved design and adoption of innovative technologies, and (*c*) to present case study examples of medical device design that apply these approaches to bridge the gap between biomedical engineers and the contexts for which they are designing.

## 2. BRIDGING THE GAP: THE NEED FOR CONTEXTUAL INVESTIGATION IN HEALTH TECHNOLOGY DESIGN

**Figure 1:**
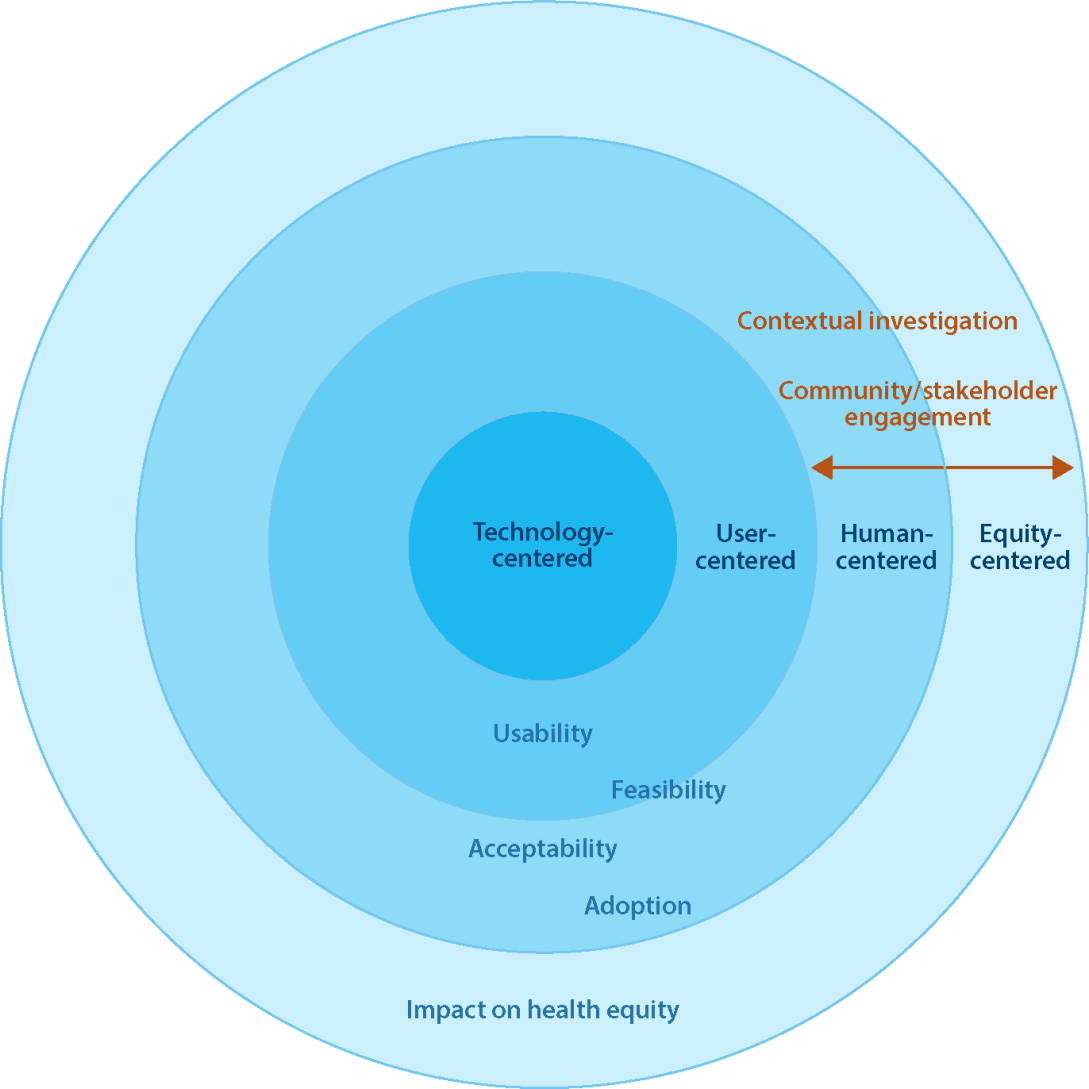
Thinking beyond technology- and user-centered approaches toward human- and equity-centered design requires meaningful contextual investigation and engagement of broader stakeholders and communities. While technology- and user-centered approaches can facilitate the usability and feasibility of an innovation, human- and equity-centered approaches can also increase its acceptability and adoption and ultimately have a greater impact on health equity.

Biomedical engineers have begun to think beyond technology-centered or technocentric design approaches to embrace user-centered approaches and involvement of end users to improve the usability of technologies and client-device interactions. However, a focus only on a user’s engagement with a final product and the technical factors closest to the device is insufficient to bridge the translation and implementation gaps (21) (Figure 1). Technology developers must understand the context in which they hope to introduce an innovation and how the characteristics of the technology will interact with this context and its broader stakeholders. Importantly, expanding the focus from end users to stakeholders is a key first step to identifying these imperative broader considerations. Prior work has identified many groups of stakeholders in medical device design including financial decision-makers, gatekeepers, customers, primary users, active users, passive users, proxy users, beneficiaries, community leaders and peers, and expert and community advisory groups (14). These stakeholders hold influence across design and implementation processes that include supply chain management, manufacturing, government, regulation, marketing, and technology adoption. In particular, broadening stakeholder engagement and contextual investigation is critical to understand the nontechnical factors, those historically considered as further from the device, including the social, cultural, political, economic, environmental, and public health contexts. These further factors, while often overlooked when informing design specifications, are the issues that most often create barriers to implementation and adoption of new technologies or, more tragically, lead to the design and implementation of harmful devices that exacerbate health inequities (24–26).

Diffusion of innovations theory (27) provides a useful framework to understand the process by which an innovation is adopted over time among the members of a social system. It describes five characteristics of an innovation that determine its adoption and rate of diffusion:(*a*) relative advantage, (*b*) compatibility, (*c*) complexity, (*d*) trialability, and (*e*) observability. The relative advantage of an innovation is how it compares to the technology or idea that supersedes it. Thus, technology designers must first understand how people are currently addressing the relevant issue and the needs, challenges, and norms of the social system prior to designing a solution. Compatibility is the degree to which an innovation is perceived as consistent with the existing values, past experiences, and needs of potential adopters. Incompatibility of an innovation can hinder its adoption; thus, designers must ensure that an innovation is acceptable and compatible with sociocultural values and beliefs, previously introduced ideas, and client needs (see the sidebar titled Compatibility with the Intended Context). Complexity is the degree to which an innovation is perceived as relatively difficult to understand and use. A designer’s understanding of the end user’s capacity and training needs is critical to ensure usability of an innovation. Trialability is the degree to which an innovation may be experimented with on a limited basis. Engaging stakeholders in design through prototyping and other iterative processes allows an individual to give meaning to an innovation and to find out how it works in their own context and enables redesigning so as to customize the innovation more closely to the individual’s conditions. Observability is the degree to which the results of an innovation are visible to others. Thus, technologies must achieve their intended purposes and outcomes within their target context and not just achieve laboratory-based performance metrics. Importantly, dissemination of findings through meaningful community engagement is critical to adoption of innovations.

Strategies to mitigate barriers to adoption can influence the core design of the technology; thus, engaging end users, communities, and broader stakeholders to understand contextual factors is a critical step to undertake at the early stages of device design. Ideally, before designing a technology, engineers should seek a clear understanding of the problem definition, including technology, stakeholders, contextual setting, use case, purpose, and operational characteristics and goals (29). The Five Ws and How, basic questions used in information gathering and journalism, are also quite useful to guide design thinking when considering broader contextual factors:

1. **What**: Is the technology actually needed and desired? If so, what characteristics of the technology are necessary and appropriate for the context of interest?
2. **Who**: Who is the appropriate or ideal end user in a given context? Often, biomedical engineers assume a clinician or other trained healthcare worker will be the end user, which may not necessarily be the case in many contexts where a lay community health worker, caregiver, peer, or the patient themselves may be operating the device or have input into the choice of solution used (see the sidebar titled Who Is the Context-Appropriate End User?). In some cases, untrained family members acting as caregivers are the primary users of at-home therapeutic technologies (30). Understanding the needs and experiences of the intended end user, the intended beneficiary (i.e., patient), and other key stakeholders is essential to inform device goals and requirements. Different stakeholders require different strategies for engagement (31) and may also have certain characteristics, for example, beliefs, values, and educational backgrounds, that set the expectations of what the product is and how it can achieve its goals effectively and efficiently (5).
3. **Where**: In which setting(s) or physical environment(s) will the technology be used? These can vary from a hospital, community clinic or health post, or mobile van to homes, nature, or other settings that require different technical specifications and levels of resource availability. Moreover, differences in settings often require consideration of context-specific design methods and approaches (e.g., for a stakeholder’s working or living space, virtual engagements, or a simulated environment) (14).
4. **When**: What is/are the appropriate use case scenario(s) of the technology? When in a workflow, healthcare continuum, or disease stage is a device to be used (33–35)? A clear understanding of the use case scenario is necessary to inform requirements and later implementation stages.
5. **Why**: What is the purpose of the technology? What is the intended outcome? Understanding the goal of the technology enables purpose-driven design and allows stakeholders to measure and monitor its impact on intended outcomes.
6. **How**: How will the technology and user interact? Technology–user interactions and usability define how the user experiences the technology (5). This interaction should inform key technology characteristics such as user interface, compatibility requirements (e.g., with electronic health records or mobile phones), result readout, time to result, packaging, instructions, etc.

To meaningfully explore and answer these questions, engagement of diverse stakeholders and communities is required. While significant barriers exist for engineers to be able to do so, including time, funding, and training limitations, it is important to recognize that engagement can take many forms and that it exists along a spectrum (36). On one end, simply identifying and consulting a few key stakeholders such as a clinician collaborator or a few patients can take a few hours. On the other end are more involved and time-intensive engagement strategies such as co-design approaches and community-based participatory research (CBPR) that provide richer contextual details and understanding. While the most involved participatory approaches may not be necessary or even appropriate, adequate engagement that allow designers to fully capture end-user and broader stakeholder and community needs is essential. Furthermore, interdisciplinary teams and collaborations with researchers in public health, anthropology, and other social sciences can facilitate and strengthen contextual investigation and leverage existing networks and deeper relationships with communities.

## 3. HUMAN- AND EQUITY-CENTERED APPROACHES TO CONTEXTUAL INVESTIGATION AND HEALTH TECHNOLOGY DESIGN

A variety of design processes and methods emphasize understanding and incorporating the context-specific needs of end users, their communities, and broader stakeholders into design decisions. Herein, we review existing frameworks and human- and equity-centered approaches to contextual investigation for improved adoption of innovative technologies, and we present case study examples of medical device design that apply these approaches to bridge the gap between biomedical engineers and the contexts for which they are designing.

### 3.1. Contextual Investigation

Contextual factors are elements of a technology’s broad context of use that could affect how that technology would be implemented and used in practice—the social, cultural, political, and economic factors that influence its use; the local resources and skills that might be available to maintain it; and how the solution is affected by infrastructure, institutions, and policy. Aranda-Jan et al. (5) present a framework for organizing contextual factors into nine categories: institutional, industrial, technological, infrastructure, geography/environment, economic, political, public health, and sociocultural. Designers that engage in the following methods are likely to incorporate these broader contextual factors: observing the context throughout their design process, actively engaging with stakeholders to collect and synthesize information, intentionally incorporating identified factors into requirements, and testing prototypes within the context and/or with stakeholders (21). In another analysis, Jagtap (37) identified five critical contextual aspects for designing solutions for marginalized populations, suggesting that designers deeply investigate the roles that the following aspects play in a design process: (*a*) user income, (*b*) urban versus rural setting, (*c*) design sector, (*d*) country, and (*e*) gender.

Studies of engineering practice have identified that professional engineers investigate contextual factors and sociotechnical considerations broadly to identify relevant information to consider in their design processes (38, 39), which Leydens & Lucena term as listening contextually (40). Indeed, reviews have argued that developing a holistic understanding of the context is a critical component of designing innovations, particularly for marginalized communities (41), that involves collecting and analyzing relevant contextual information primarily through ethnographic methods such as observations, interviews, and surveys during early stage problem identification and backend feasibility pilot studies in the intended context of use (42). In a recent study of global health design practitioners, participants described consistently and extensively considering broad contextual factors throughout their design processes (G. Burleson, K. Toyama & K. Sienko, unpublished manuscript). For example, they engaged a broad range of stakeholders to understand context, and they visited and revisited contextual information, adjusting the scope of their projects, even to the point of terminating a project, based on the contextual information they gathered and analyzed. However, studies have demonstrated that engineering students consider and incorporate contextual factors in much more narrow ways, focusing on technical considerations to a far greater extent than broader social considerations (39, 43, 44). It is important to note, however, that students’ desire to incorporate broader social considerations has been shown to exceed the course structure and tools provided to them (21).

### 3.2. Human-Centered Design

Human-and user-centered design approaches focus designers’ attentions on end-user needs, experiences, and contexts of use. User-centered design methods aim to enhance end users’ interaction and engagement with the final product, emphasizing improvements in safety and usability (45). Thinking beyond the direct user, human-centered design more explicitly seeks to integrate an innovation into human activities and systems by considering individuals beyond primary users in a design process, including those who interact indirectly with the innovation, such as clinic leaders who oversee implementation, as well as those who are unintentionally affected by it, such as family members of patients and broader communities (41, 46, 47). While user-centered design focuses on end-user experience, human-centered design encourages designers to identify broader stakeholder needs by emphasizing empathy-building activities, prototyping techniques, and regular feedback loops with stakeholders throughout various stages of design processes. In particular, human-centered design has been advocated for use in global health applications due to its prioritization of stakeholders’ needs and lived experiences (4, 48).

Human-centered design emphasizes building empathy, particularly with end users, to better understand their experiences, contexts, and true needs. To build empathy, Kouprie & Visser (49) present a four-stage framework that includes phases of (*a*) discovery, (*b*) immersion, (*c*) connection, and (*d*) detachment, emphasizing working and researching in the context of use. A study by Morris & Cormican (50) concluded that empathy-focused design methods could be applied in the medical device industry in Ireland to effectively identify user needs and expectations. However, it is important to note that practicing empathic design is particularly difficult when working across cultural contexts. A study by Li et al. (51) measuring the empathic accuracy of designers engaging with stakeholders found that national cultural differences significantly affected the accuracy of designers’ empathic understanding of the population for whom they were designing. Since an individual’s ability to empathize is inherently determined by their beliefs and judgments, designers may believe themselves to be practicing empathy in design while instead perpetuating their own biases and stereotypes. As such, empathic design requires critical self-reflection to gain a deep understanding of what information and values a designer may be selecting versus neglecting (52) and to examine their positionality and its potential effect on design decision-making (52a).

In the literature, there are many examples of engineers applying user- and human-centered design processes in medical device development across design stages. Human-centered design processes have been used to develop tools for patients with multiple sclerosis (53) and to design neuroprosthetics and exoskeletons (54). To evaluate early concepts to improve the design of a hospital bed, Wiggermann et al. (55) conducted focus groups and usability tests with more than 130 users. Other case studies emphasize human-centered usability tests to evaluate diagnostic devices during prototyping stages of design (56, 57). These types of formative usability studies have helped to counteract assumptions about healthcare provider capabilities during task shifting. For example, a study by Mohedas and colleagues (58) highlighted the importance of selecting participants for usability studies, suggesting that proxies may not necessarily reflect performance of the intended end users.

In healthcare more specifically, patient-centered design aims to incorporate the principles of patient-centered primary care, including improved access to care, patient engagement in care, quality improvement, care coordination, smooth information transfer, routine patient feedback, and transparency (59), into engineering design processes for clinical applications. Chao et al. (60) advocate for patient-centered approaches to medical device design, aiming to put the needs of the patient at the forefront of any competing interests. Designers have used patient-centered design to develop technologies that support patients in their homes (61, 62). In hospitals, health providers have ranked facilities that emphasize patient-centered design higher than those that do not, in terms of improvements in safe and efficient care (63). However, many medical solutions are not developed with these priorities. While beneficence, helping patients, and nonmalfeasance are always the end goal, often the convenience of the innovator and health provider and profitability are prioritized over the autonomy of the patient or justice in equitable access (64).

Human-centered design approaches have historically been more common in the computing and information technology fields, and medical device design can benefit from the rich experiences of technology companies building human–computer interactions and user interfaces. Numerous examples of interdisciplinary collaborations and frameworks for evaluations of human–computer interaction prototypes as well as case studies exist in this space (65–67). These design frameworks have been applied to mobile health (mHealth) in recent years to improve user interaction experiences. Of note, a 2016 meta-review by Krah & de Kruijf described issues with adoption of earlier versions of mHealth technologies in Africa due to the attempt to design applications with overly broad scope and the developers’ “insufficient understanding of beneficiaries and specific context of use” (68, p. 1). These same issues apply to the field of medical device design more generally. To combat these challenges, human-centered design from formative to backend evaluations can improve both development and adoption of technologies.

#### 3.2.1. Case study.

Cornet et al. (69) designed an application for older adults with heart failure and integrated user expertise across the formative research, design, and evaluation stages of their work. At the formative stage, patient interviews, advisory meetings, and meetings with clinician advisors were incorporated to establish the problem scope and design requirements to support older adults with a cardiac implantable electronic device. At the development stage, designers generated prototype user-interface dashboards and worked with user participants to determine the preferred information flow and layout. Three groups of three individuals, consisting of a mix of elderly patients and informal caregivers, provided feedback at the prototype stage of the design. Lastly, usability and heuristic evaluations with patients and user-centered design experts informed final development stages (69).

#### 3.2.2. Takeaway.

Throughout the process, the author designers took proactive steps to achieve innovation equilibrium by involving diverse and representative stakeholders early, managing designer assumptions via validation and disconfirmation with stakeholders, managing stakeholder heterogeneity, accommodating clinical workflows and regulatory limitations, and balancing the desire to create overly complex designs for the sake of innovation with the practical user needs for simplicity (69).

### 3.3. Design Ethnography

Designers who use human-centered design methods advocate for field research and the use of design ethnography techniques throughout design processes, such as observations and in situ interviews, but particularly during early problem definition phases to collect key information about stakeholders and their context (70, 71). The use of iterative design ethnography techniques, including collecting regular feedback by visiting and observing the use context often, is emphasized in applications of human-centered design in medical device development (72). Design ethnography allows for the investigation of both the technical usability factors closer to the device as well as further contextual factors that inform its implementation.

#### 3.3.1. Case study.

Sabet Sarvestani & Sienko (73) describe the applications of design ethnography to develop a culturally appropriate medical device that would be appropriate for both traditional male circumcision (TMC), which is a rite of passage into adulthood, and voluntary male medical circumcision, an effective HIV prevention method that requires complete removal of the foreskin (74). The initial concept and prototype were designed as part of a capstone project with input from literature and clinical experts. The preliminary concept, developed by a team of capstone design students, met the initial requirements and specifications; however, given the dearth of publicly available data about TMC practices, the initial requirements and specifications were solely informed by benchmark analysis of existing pediatric circumcision devices (primarily used in a clinical setting) and by interviews with and observations of US-based physicians who performed pediatric circumcisions. Following the award of a Gates Foundation Grand Challenges Exploration grant, author designers traveled to Uganda and completed numerous semi-structured interviews and focus groups and TMC observations, leveraging principles of design ethnography to better understand the broad patterns of daily life that pertained to TMC across multiple ethnic groups (73). These data were used to inform substantial changes to the requirements and specifications (Table 1).

#### 3.3.2. Takeaway.

As the authors describe, “The techniques were key to establishing and confirming the need, which had a significant cultural load associated with it. They also helped us understand the stakeholders’ viewpoints and concerns, and provided data used to generate justifiable user requirements and associated engineering specifications” (75, p. 7). Although cost was important, they learned that it was not sufficient to ensure uptake and that cultural norms would likely greatly affect the adoption and sustained use of such a device; for example, the assumed initial requirement of “one size fits all” was not well received because numerous stakeholders who were engaged, including the traditional cutters and assistant cutters, did not trust the device’s ability to accommodate all sizes (instead they requested that the devices be manufactured in multiple sizes and provided t-shirt sizing as an example). They also learned about the important roles of religious leaders, churches, and mosques in promoting and disseminating such interventions and of workers’ unions in preserving TMC’s cultural significance.

### 3.4. Engaging Stakeholders with Prototypes

Stakeholder engagement, particularly with prototypes, is advocated in medical device literature, particularly during early stages of problem definition and requirements development (14). In design fields that focus on digital health solutions, stakeholder engagement is especially encouraged (76, 77). Interviews, questionnaires, workshops, focus groups, and observations are highly cited methods for engaging with stakeholders (78), particularly at key decision-making stages during design processes (4). In response to the coronavirus pandemic, Antonini et al. (79) developed the crisis-responsive design framework for medical device development under pandemic conditions; the framework utilizes extensive stakeholder engagement and comprehensive needs assessment.

Specifically, engaging stakeholders with prototypes has been shown as a valuable method for uncovering contextual information and stakeholder needs (80). Scholars have characterized the use of prototypes during stakeholder engagement in early stages of medical device design, identifying 17 unique strategies used by medical device designers across both high-income and low- and middle-income settings (81, 82). Furthermore, a recent study described the breadth of prototypes (physical 3D,2D, and digital 3D) used with stakeholders (users, implementation stakeholders, and expert advisors) across various settings including meeting space, simulation environment, real-use environment, and distant settings (i.e., virtual) to collect relevant user and contextual data to inform design decisions (14). Studies of novice designers in the United States and Ghana have revealed that engineering students use some recommended practices to engage stakeholders, such as using prototypes to communicate, test, and identify functional blocks, but reveal that their prototyping during early stage problem scoping is underutilized and often executed in unstructured and unintentional ways (83, 84).

#### 3.4.1. Case study.

Linnes et al. (56) describe the use of a product-hypothesis-generated test for field trials with regard to the need, users, context, and critical assumptions for a prototype sickle cell diagnostic tool during early evaluation of prototypes in Zambia. After designing initial prototypes on the basis of assumptions from literature and expert interviews, researchers traveled to Zambia to shadow clinic staff and perform in-context interviews. They determined that the primary users would be community health workers and paramedical staff rather than the originally assumed nurses, that the time to result could be extended to 30 min rather than only 20 min, and that more resources were available than assumed, including basic microcentrifuges and 12-V car batteries for power (56) (Table 2).

#### 3.4.2. Takeaway.

Engaging stakeholders in context is critical for identifying potential challenges and testing assumptions. Performing an even earlier in-context evaluation during the formative stages of the design process could have further reduced the need to overconstrain early prototypes to power-free designs and ensured usability for staff with less formal medical education.

### 3.5. Concept Target Product Profiles

A target product profile (TPP) is a strategic document that lists desirable characteristics of a product, such as the minimal and optimal performance and operational features of diagnostic tests. TPPs are meant to guide research and development and, as such, contain sufficient detail for technology developers to understand the requirements for a product to be successful. This includes not only technical requirements but also features that allow use in a defined setting, including safety- and efficacy-related characteristics. TPPs are developed when the use cases are already defined and it is known when, where, and why the technology will be used. When available, a TPP is an ideal starting blueprint for technology designers.

When a TPP does not yet exist and the use case is not yet well defined, the development of a concept target product profile (CTPP) can be a useful tool to help engineers identify key contextual factors and specific needs earlier in a design process (33, 34). A CTPP defines the context-specific need via the Five Ws and How of the use-case scenario that the medical device solution should address, and informs the technical requirements of the solution. While TPPs tend to narrowly focus on the How alone, the CTPP provides important context that can aid in concept solution selection. For example, when deciding between home health monitoring solutions, the context of the frequency of use becomes an important factor in guiding designers in their efforts to determine whether users would be willing to store a reusable item between uses or would prefer to purchase a disposable item. Overall, a CTPP can serve as a helpful first step toward creating a TPP when one does not yet exist.

#### 3.5.1. Case study.

**Figure 2:**
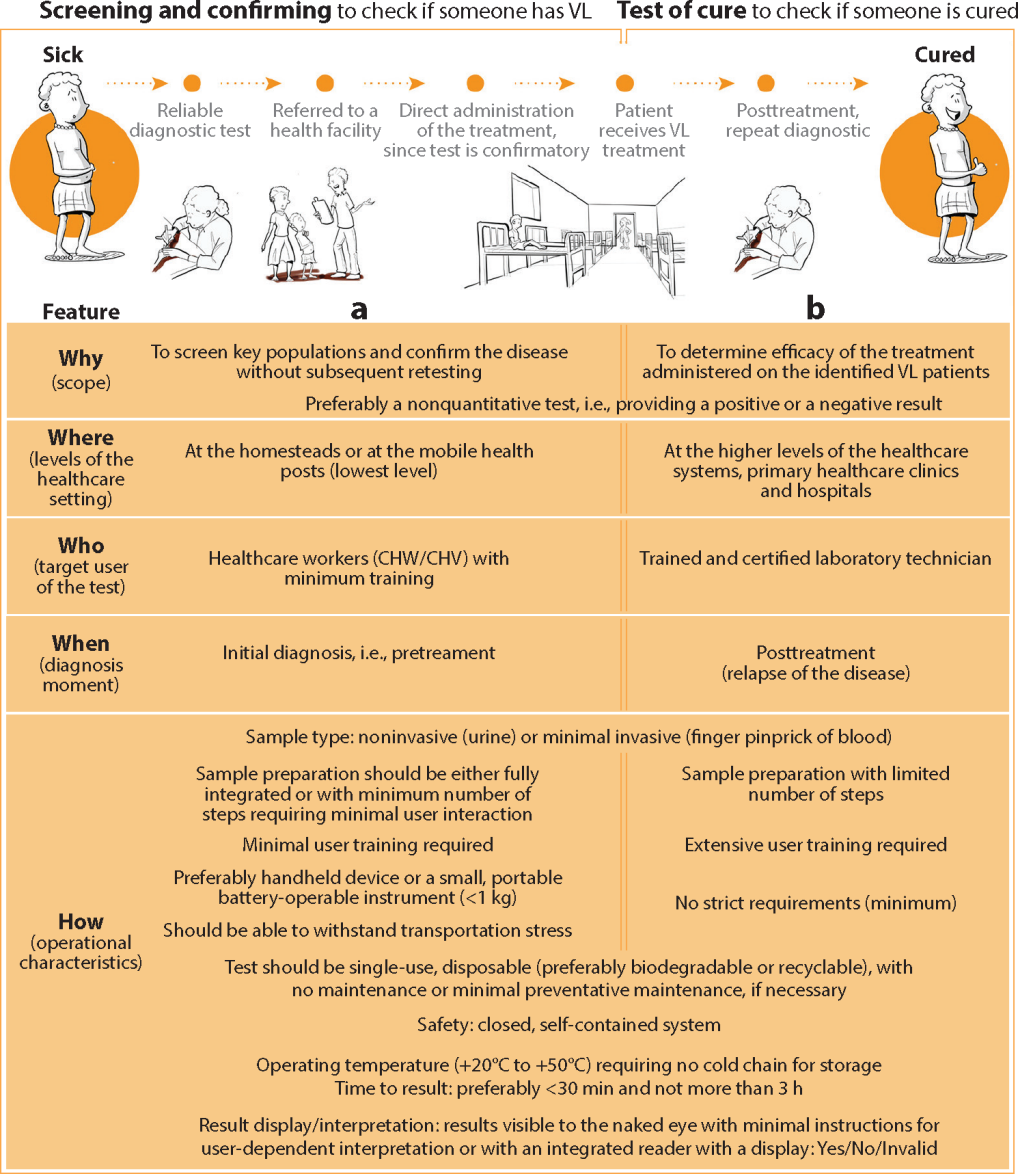
In the absence of a TPP for a point-of-care diagnostic test for VL, Bengtson et al. (33) engaged diverse stakeholders to understand context-specific needs and defined a CTPP to guide early design efforts. Abbreviations: CHV, community health volunteer; CHW, community health worker; CTPP, concept target product profile; VL, visceral leishmaniasis. Figure adapted from Reference 33 (CC BY 4.0).

Bengtson et al. (33) describe a process for developing a CTPP to guide early design efforts for a point-of-care diagnostic test for visceral leishmaniasis, for which a TPP did not exist. They used design-thinking principles to evaluate diagnostic processes, identify specific needs, and determine appropriate technological solutions. They conducted observations and semi-structured in-context interviews with healthcare providers, patients, local officials, and community members to gain empathic understanding of the problem. They diagrammed patient journeys from infection to treatment considering the stages, locations, costs, providers, barriers, and care at each stage to create and validate use-case scenarios and ultimately define the CTPP (Figure 2).

#### 3.5.2. Takeaway.

In the absence of a TPP for a diagnostic test or other identified technology of need, engineers can engage diverse stakeholders to understand context-specific needs and to define the Five Ws and How of the target technology in a CTPP.

### 3.6. Participatory Design and Co-Design

Participatory design emphasizes a closer partnership between designers and stakeholders, particularly in decision-making stages throughout design processes. This direct involvement of stakeholders as co-designers throughout a design process emphasizes their expertise, experiences, values, and context (85). In the majority of design processes, stakeholders are passive participants who receive a solution rather than active participants in the generation of one; thus, there is an opportunity for more co-design methods and frameworks, such as collective deliberation (86), in medical device innovation (87). Various participatory design methods (e.g., cultural probes, personas, focus groups, workshops) have been suggested for medical device design (88), especially in low- and middle-income settings when there are often cultural, social, and political differences between designers and users (89). A specific type of participatory design that is frequently recommended when working with marginalized communities is co-design, which is a socially embedded approach that directly involves communities in decision-making throughout a design process, encouraging necessary incorporation of their preferences and context(90,91). Co-design approaches allow designers to look beyond solely technological aspects of product design to the social and cultural context of marginalized communities, particularly as it relates to their empowerment and values by extending decision-making power (92).

These participatory design methods are minimally applied in medical device design, and there are only a handful of examples in the literature of their application, such as case studies of using participatory design to develop medical robots (93) and electronic implants (94).

#### 3.6.1. Case study.

**Figure 3:**
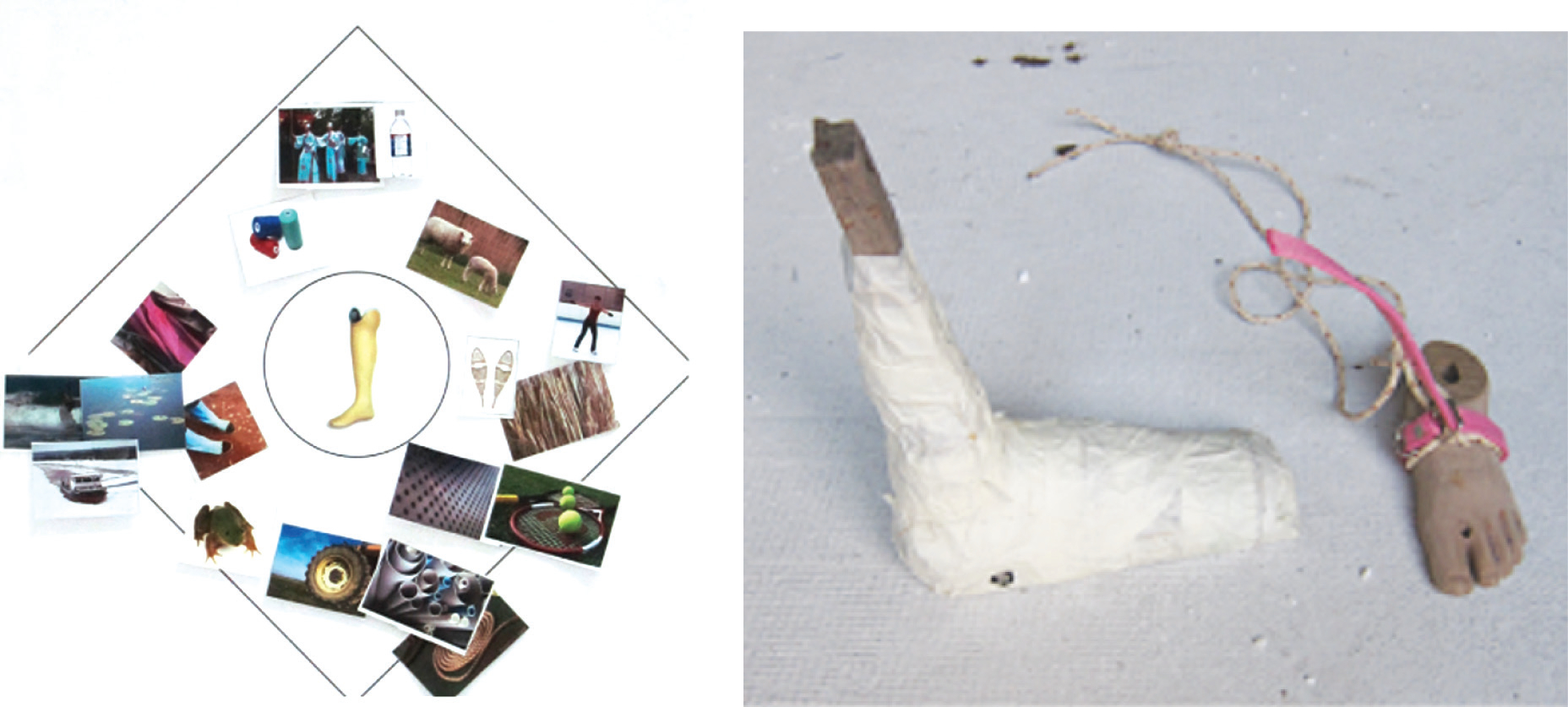
Co-design process of a prosthetic device for children in rural Cambodia. (*Left*) Co-design participants selected images to inspire the design of their desired product, including factors related to functionality (e.g., lightweight, waterproof) and aesthetics (e.g., brightly colored). (*Right*) An early prototype co-designed with diverse stakeholders that included a string attached to the foot that could be pulled if the prosthetic became stuck in the mud (a key concern among end users). Figure reproduced from Reference 96 (CC BY-NC-ND 4.0).

Hussain & Sanders (95) applied co-design methods to design a prosthetic device for children in rural Cambodia to enable them to walk in mud during the rainy season (Figure 3). The author designers conducted interviews with children, parents, and other key stakeholders in the context including Buddhist monks, traditional medicine men, adults who had been using prosthetic legs since childhood, and rehabilitation workers; the authors also observed patients at rehabilitation centers as well as the production of prosthetic components. Doing so allowed the researchers to obtain an initial understanding of the main usability issues, cultural health beliefs, and social implications of prosthetic use. This was followed by the development of generative design tools to seek a deeper understanding of how children are affected by using prosthetic legs and how changing the appearance of the prostheses can benefit children. Then the team led a series of workshops with designers, users, stakeholders, and prosthetists working together to generate designs and received iterative feedback from the children along the way. The field research resulted in positive outcomes of two types: a product that met the end users’ needs and the empowerment of the participants in a historically marginalized and underserved setting.

#### 3.6.2. Takeaway.

Participatory design provides an opportunity to empower participants to co-create their own solutions, while developing products that meet real-world and context-specific needs.

### 3.7. Community-Based Participatory Design

CBPR is one of several approaches to research in the health and social sciences with long-term engagement of communities in all aspects of the research process—from identification of priorities, formulation of research questions, study design, data collection, interpretation, and dissemination of findings to determination of action and policy implications (97, 98). This co-led approach to research is time and resource intensive and depends on the mutual interest of academic and community partners. Therefore, while CBPR is not always a feasible approach, it can have powerful benefits for both communities and technology developers. CBPR has been shown to improve the rigor, relevance, and reach of science (99), by bettering the appropriateness of technology and intervention designs, improving the quality of data collected, facilitating participant recruitment, addressing issues of relevance to community members, and building trust and partnership between researchers and community members. The benefits that CBPR generates for community partners have been well documented and include enhancing community empowerment, co-learning between community members and scientists, informing community organizing efforts, and linking research to policy action.

While CBPR has primarily been used in public health and environmental health sciences, these community-based participatory approaches are being explored in engineering for contextual investigation to inform more meaningful design of both technologies and their implementation strategies. Engineering and public health researchers have begun integrating CBPR and human-centered design approaches to better understand the needs and experiences of end users, along with their broader social and structural contexts, and to co-develop health interventions (100). Both CBPR and human-centered design are people-centered approaches to addressing real-world problems, and combining them can lead to more effective, scalable, and sustainable solutions (101).

#### 3.7.1. Case study.

Cervical cancer screening rates are declining in the United States, particularly among uninsured and medically underserved communities. A rapid human papillomavirus (HPV) test could potentially address well-documented barriers to Pap smears and laboratory-based HPV testing. Toward this end, a prototype rapid HPV test achieving technical metrics of sensitivity and specificity was developed (102), but without meaningful engagement of end users or contextual investigation. Upon approaching an underserved community with excess cervical cancer burden to explore the potential for device feasibility studies, engineers learned of key social, cultural, political, and economic contextual factors that would prevent the successful adoption of the technology (103). For such a technology to have meaningful impact on cervical cancer screening rates and health outcomes, contextual investigation is necessary to inform device redesign and define key implementation strategies. A CBPR study, funded by the National Cancer Institute, is ongoing and is engaging diverse stakeholders including community members, patients, clinicians, community health workers, and policy makers in a participatory innovation process to inform the Five Ws and How of a rapid HPV test to address cervical cancer screening disparities (104, 105, 105a).

#### 3.7.2. Takeaway.

Integrating CBPR and human-centered design into a medical device design process in research and industrial labs could lead to the development of better solutions to meet not only the technical specifications but also the user implementation needs for impactful solutions and to reduce costly redesign.

### 3.8. Design Justice and Equity-Centered Design

Design justice and equity-centered design prioritize the needs, experiences, and perspectives of the most marginalized individuals and communities within a particular context, ultimately aiming to liberate communities from exploitative and oppressive systems while sustaining and empowering them (106). Notably, these approaches view the role of the designer as a facilitator rather than an expert and prioritize the design’s impact on the community over the intentions of the designer (107). In these approaches, understanding the context of design is key to being able to identify existing inequities that can be addressed as well as the theoretical framings to use during design. Equity-centered design scholars emphasize using critical theory lenses, such as critical race theory (108) and intersectional feminist theory (109), which are inherently context specific and apply to specific design processes on the basis of the historical and current systems of oppression in a particular setting. For example, using design-justice principles, Zidaru et al. (110) identified specific challenges and opportunities associated with artificial-intelligence-assisted mental healthcare. Furthermore, using a design-justice approach in a global health context emphasizes consideration of historical and underlying economic frameworks, particularly since many engineering projects are situated within contexts of colonial legacies and neoliberal economic and geopolitical policies (111).

## 4. DISCUSSION

The approaches and case studies presented in this review are intended to help guide and motivate biomedical engineers toward meaningful stakeholder engagement and contextual investigation to improve technology design in teaching, research, and practice. A shift in the traditional technocentric view of medical device design to a human-centered one is needed to bridge existing translational gaps and ensure the successful and equitable uptake, adoption, and diffusion of biomedical innovations to ultimately improve health equity (Figure 1). A stronger emphasis in our field on a broader understanding of context during early design stages, the importance of diverse stakeholder perspectives, and the need for iterative participatory design processes will lead to improved outcomes in real-world settings.

As a field, we must increase both the acknowledgment and application of broader contextual and sociotechnical considerations during the design of medical innovations. Projects regularly fail when they neglect to recognize the social and institutional complexity of the environment in which the product is deployed (112). Rapid changes in political environments, supply chains, and workforce availability all require the designer’s attention, since these factors directly influence the design, implementation, and success of healthcare innovations (26). Moreover, advancements in technology over the recent decades continue to require more complex technological considerations, including increasing role of telemedicine, connectivity, and automation in the design of medical devices (25); the success of such technological implementation inevitably relies on its suitability and appropriateness within a given context of use. Overall, engineers must acknowledge and account for the technical, social, and political processes that enable or constrain effective implementation and use in their design. Furthermore, engineers should consider their positionality, which affects inclusive design approaches, relationships (e.g., power dynamics between themselves and stakeholders), and the influence of their own values and biases on their design decisions (113).

Biomedical engineers historically have been taught to think about contextual factors relating to the needs of direct end users and competitive products within a regulatory environment. These pedagogical perspectives are required for accreditation by ABET (https://www.abet.org/accreditation/) and are included in undergraduate design courses, such as Biodesign: The Process of Innovating Medical Technologies at the Stanford Graduate School of Business (https://www.gsb.stanford.edu). However, engineering design courses often omit broader social, economic, political, and cultural contextual factors that strongly influence whether or not a technology is ultimately used and adopted. Unlike traditional engineering design courses, stakeholder engagement and contextual factors are often brought into global health design courses, particularly those with immersion opportunities abroad, such as the University of Michigan’s Global Health Design cohorts (114) and Rice University’s Rice360 Global Design teams (115). These programs, among others, emphasize co-creative design processes involving local stakeholders and intentionally partner students from different contexts together to collaborate on design projects (116). While educational immersive experiences abroad are difficult to provide at scale, contextual investigation is inherent to training programs in sociology, anthropology, public health, and other social sciences as an integral part of curriculum and do not innately require resource-intensive activities or study abroad. Presently, students interested in contextually appropriate, equity-centered design are expected to supplement their technical BME learnings with elective courses in other departments that do provide training in contextual investigation, rather than such training being integrated in BME pedagogy as a fundamental skill of engineering design.

Real consideration of who we are designing for, and understanding of their broader societal contexts, must not be limited only to global health applications. While many of the case studies presented herein and elsewhere focus on low- and middle-income contexts, the approaches, methods, and frameworks described are applicable and needed across all settings. Although the historical and contextual differences that influence outcomes in health vary across settings, health inequities are prevalent across all resource and income levels. A lack of technological innovation in contextual investigation can be a substantial driver of these inequities in several important ways. When resulting technologies are costly or designed to require complex infrastructure, they may improve health outcomes in high-resource settings while remaining largely inaccessible to lower-income populations, further widening disparities. Additionally, when social contexts are ignored in health technology design, the negative consequences are generally experienced by the most vulnerable (117). An important example highlighted by Fawzy et al.(118) in 2022 is that of finger clip pulse oximetry devices that were particularly widely used and critical during the COVID-19 pandemic. Because these optical technologies had been designed and evaluated with predominantly White patients, their accuracy was significantly flawed among Asian, Hispanic, and Black patients because of discrepancies in oxygen level measurements across darker skin pigmentation. Critically, this led to delayed and suboptimal care, and worse COVID outcomes, particularly for Black patients. Of note, the overall accuracy of US Food and Drug Administration (FDA)-approved devices remained within the FDA guidelines of ±3% overall root-mean-squared error accuracy (118a), but inaccuracies resulted in inequitable outcomes on the basis of the color of an individual user’s skin. A better acknowledgment of the historical inequities that have led to lighter-skinned people receiving more care and attention, appreciation for the biases that affect both design and testing decisions, and awareness of racial inequities in the context of medical care decision-making could have led designers to increase prioritization of accuracy among darker skin tones and yielded better outcomes for marginalized patients in the United States.

Finally, without adequate stakeholder engagement and contextual investigation, the actual real-world needs and priorities of communities can remain largely overlooked. For example, needed technological innovation for neglected tropical diseases, which affect more than one billion people globally, continues to be underprioritized and underfunded (119). Even in high-income settings, conditions such as endometriosis, despite affecting 7 million women in the United States and 200 million worldwide, continue to be underresearched, resulting in a critical lack of tools for timely diagnosis and treatment (120). There is also a substantial mismatch between the increasing number of people affected by noncommunicable diseases globally and the relevant number of commercialized medical devices designed specifically for use in low-income countries; furthermore, only a limited number of commercialized devices have been designed specifically for use by nonphysician health providers (24). By engaging communities and refocusing our pedagogical and research efforts toward equity-centered engineering with meaningful consideration of context, biomedical engineers can move the needle toward the development of technologies that are adopted and used in ways that lead to greater equity in social and public health outcomes.

## 5. CONCLUSION

The success of an innovation is determined not by how well it meets technical specifications but by its implementation, adoption, and ultimate impact in its intended social context. To ensure the success of health technologies being developed, engineers must think beyond the technology and end user and seek a more holistic understanding of the context of use early in the design process. To do this appropriately, designers must meaningfully engage communities and other stakeholders more broadly to understand not just how a technology will be used but also the social, cultural, political, economic, and environmental factors that may influence its adoption in a given context. Biomedical engineering training, research, and practice must look beyond the traditional technocentric focus on technical specifications, and even beyond the more recent user-centered focus that emphasizes usability and feasibility, to a broader human- and equity-centered focus on acceptability, adoptability, and, ultimately, impact on health equity. The approaches and examples outlined in this article provide initial guidance on how to do so and are applicable and needed not just in global health or low-income settings but in all settings and applications of designing technologies for people.

## Figures and Tables

**Table 1:** Original and revised user requirements and corresponding engineering specifications for a traditional male circumcision device [adapted from Sabet Sarvestani & Sienko (73)]

Original requirements	Original engineering specifications	Revised user requirements	Revised engineering specifications
Fast cut	120 sec	Fast cut	<10 sec
Safe cut	50% glans coverage	Safe cut	100% glans coverage
Number of parts	3	Strong grip	No displacement while cutting
Adjustable diameter	1.5–4.1 cm	Multiple sizes	Small (2.5 cm), medium (3.0 cm), large (3.5 cm)
Number of steps	10	Low cost	Final cost <$1

**Table 2:** Initial and revised device use cases for a prototype sickle cell diagnostic test [adapted from Linnes et al. (56)]

Initial device need and use case	Revised device need and use case
Nurses at rural health centers in Zambia need a <$1 diagnostic test for sickle cell disease to screen newborns and identify common genotypes that takes less than 20 min to perform without additional power	Community health workers and paramedical staff at rural and urban health centers in Zambia need a <$1 diagnostic test for sickle cell disease to screen children at the time of routine vaccinations that is simple to interpret and takes less than 30 min to perform with up to 12-V power sources

## COMPATIBILITY WITH THE INTENDED CONTEXT

In the 1970s, Rogers & Pareek (28) carried out studies commissioned by WHO to understand the causes of low rates of adoption of contraceptive methods and the ideal attributes of family planning technologies. They found that many communities in low- and middle-income countries were averse to contraceptives that required any kind of genital handling (e.g., condoms, intrauterine devices, diaphragms), which were the main methods of family planning promoted at the time. This understanding led to the design of more socially acceptable technologies such as injectable and implantable contraceptives.

### Takeaway

The compatibility of biomedical innovations by the intended end users, communities, and contexts is critical to their adoption. Contextual investigation and understanding of end-user acceptability early in the process of innovation can guide and prioritize research and development activities on the basis of what kinds of technologies would make the most impactful solutions.

## WHO IS THE CONTEXT-APPROPRIATE END USER?

End users of malaria rapid diagnostic tests are likely to be remote health workers, often volunteers with limited training. The evaluations of new tests are commonly carried out by highly trained technicians in controlled laboratory environments and therefore differ significantly from that of the intended end users. Performance of the test and interpretation of results can also be affected by manual dexterity, visual acuity, and available lighting. Studies have documented significant variation between trained technician evaluators and lay health workers (actual end users) in both rapid diagnostic test preparation and interpretation (32).

### Takeaway

An understanding of who the context-appropriate end user of a given technology will be, given the specific contextual considerations, is essential to ensure optimal performance and adoption.
